# Hardwood Burning as a Dominant Source of Fine Particulate Matter from Biomass Burning in Ho Chi Minh City, Vietnam

**DOI:** 10.1021/acsomega.5c13491

**Published:** 2026-03-31

**Authors:** Ngoc Tran, Yusuke Fujii, To Thi Hien, Norimichi Takenaka

**Affiliations:** † Division of Sustainable System Sciences, Graduate School of Sustainable System Sciences, 12936 Osaka Metropolitan University, Sakai 599-8531, Osaka, Japan; ‡ Faculty of Environment, Ho Chi Minh City University of Natural Resources and Environment, Ho Chi Minh City 700000, Vietnam; § Faculty of Environment, University of Science, Ho Chi Minh City 700000, Vietnam; ∥ Vietnam National University, Ho Chi Minh City 700000, Vietnam

## Abstract

While biomass burning (BB) significantly affects air quality in Southeast Asia, its contribution to PM_2.5_ in Ho Chi Minh City (HCMC), Vietnam’s most populous city, remains poorly understood. This study aimed to identify the major BB sources contributing to PM_2.5_ in urban HCMC, with particular attention to the potential underestimation caused by chemical degradation of levoglucosan (Lev), a widely used BB tracer. PM_2.5_ samples were collected from October to December 2021 and analyzed for their chemical composition, including Lev, mannosan (Man), and potassium from BB (K_BB_^+^). The average PM_2.5_ mass concentration was 26.6 ± 8.8 μg m^–3^ and was higher during the dry season than the rainy season due to reduced wet deposition. Notably, evaluation of BB tracers indicated substantial chemical degradation of Lev, with an estimated loss of approximately 88%. After correcting for this degradation, the reconstructed Lev concentration without chemical loss (Lev_no‑chem_) reached 1766 ± 637 ng m^–3^. Diagnostic ratios of Lev/Man, Lev_no‑chem_/K_BB_^+^, and OC/Lev_no‑chem_ were 16.7 ± 2.6, 1.76 ± 0.39, and 4.54 ± 0.74, respectively. Importantly, source identification based on the uncorrected Lev would have suggested crop residue and grass burning as the dominant BB sources. In contrast, the degradation-corrected ratios consistently indicate that hardwood and hardwood charcoal combustion from cooking are the predominant BB sources in HCMC. Weighted concentration weighted trajectory analysis further revealed that these emissions are primarily associated with local urban activities rather than long-range transport. These findings demonstrate that accounting for Lev degradation is essential for accurate BB source identification in Southeast Asia.

## Graphical abstract

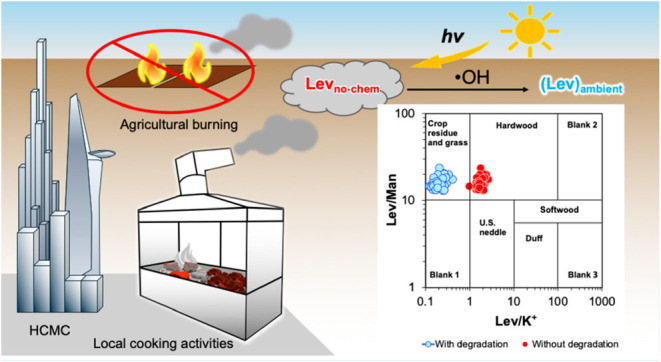

## 1 Introduction

Biomass burning (BB) refers to the combustion of living and dead vegetation, including open fires (e.g., forest, slash-and-burn of forest for agricultural uses, peatlands) and biofuels used for anthropogenic purposes (e.g., cooking, heating). BB is a substantial source of fine particulate matter (PM_2.5_),^1–3^ accounting for approximately 70% of primary PM_2.5_ on the global scale.^4^ PM_2.5_ from BB has caused severe environmental effects, such as triggering air pollution episodes, altering the Earth’s radiative balance, and accelerating global warming.^5–7^ In addition, cumulative exposure to PM_2.5_ poses a serious threat to public health, particularly during severe haze events caused by BB.^4,8,9^ Hence, controlling BB emissions, particularly through identifying the predominant types of BB, is essential for mitigating PM_2.5_ exposure and improving air quality at both regional and global scales.

Anhydrosugars, including levoglucosan (Lev), mannosan (Man), and galactosan (Gal), are commonly used as tracers for BB source identification due to their high abundance in BB smoke.^5,10,11^ Lev originates from cellulose pyrolysis at temperatures above 300 °C, whereas Man and Gal are the thermal pyrolysis products of hemicellulose, making them distinctive source-specific markers for BB types.^11^ Lev is considered an ideal tracer for BB owing to its high abundance and long atmospheric stability.^5^ Wang et al.^12^ further reported that Man and Gal are removed more rapidly than Lev, indicating their lower stability. Despite their lower stability, the relative differences among these compounds provide useful source information. As a result, Lev and its mass ratios with other BB markers (e.g., Lev-to-Man (Lev/Man), Lev-to-BB-potassium (Lev/K_BB_^+^), and OC-to-Lev (OC/Lev)) have been widely used to distinguish the combustion of hardwood, softwood, crop residues, and grass.^5,13,14^ However, after being released from fresh smoke, anhydrosugars could react with gas-phase oxidants (e.g., OH) via the heterogeneous reactions in the atmosphere.^12,15,16^ The chemical loss mechanism of particulate Lev under the heterogeneous oxidation by OH depends on particle size, particle phase and mixing state, the concentration of OH, meteorological factors (e.g., temperature, relative humidity), and reaction rate constants during the calculation.^10,15–18^ Consequently, the particulate Lev lifespan ranges from 5 min to 53 days, corresponding to time scales of deposition and long-range transport.^17^ This phenomenon presents potential uncertainties in using measured anhydrosugars to categorize the biomass fuel profile of PM_2.5_ at the receptor site.^5,10,15,19,20^ To address this issue, Li et al.^19^ developed a correction factor method to estimate the atmospheric degradation of particulate Lev (Lev_no‑chem_). This correction factor has become a methodological basis for subsequent studies in reducing the underestimation of BB and improving the identification of BB source profiles. For instance, in Changzhou, China, hardwood combustion was determined as an additional BB source of PM_2.5_, along with identified sources (i.e., crop residue burning and grass burning), in considering the Lev degradation.^21^ In Changchun, China, Shi et al.^22^ reported that, after applying the correction factor method, the contributions of wheat and corn combustion to PM_2.5_ were overestimated, while those of pine, rice, poplar, and grass burning were underestimated. Hence, these findings underscore the importance of using the correction factor and Lev_no‑chem_ as a practical approach for BB source identification in the studied region.

In recent years, BB has been evaluated as a significant PM_2.5_ source in Southeast Asia (SEA), accounting for an estimated 99% of fire-produced PM_2.5_ in Bangkok (Thailand), 50% in Kuala Lumpur (Malaysia), and 41% in Singapore,^23^ where agricultural waste and peatland are the primary biomass fuels.^8,23–25^ Although Vietnam was ranked as the second most PM_2.5_ polluted country in SEA,^26^ the primary BB source types in this country remain poorly characterized. While BB emissions and PM_2.5_ have been studied in the capital, Hanoi,^27–32^ such research is notably lacking for Ho Chi Minh City (HCMC), one of the most populous cities in Vietnam and SEA. PM_2.5_ levels in HCMC regularly exceed 20 μg m^–3^,^33–36^ posing significant health risks to residents.^34^ Our previous study reported that anthropogenic emissions (including BB, transportation, and coal combustion) accounted for 36.4% of the PM_2.5_ levels in urban HCMC.^37^ While potential BB sources contributing to PM_2.5_ in HCMC likely include local cooking activities^37^ and/or long-range transported emissions from crop residue open burning in the Mekong Delta,^33,38^ the specific BB types remain unidentified. This knowledge gap hinders our understanding of the environmental impacts of BB emissions and delays the development of effective mitigation strategies for this megacity.

To fill this gap, this study aims to identify the major BB types contributing to PM_2.5_ in urban HCMC, with particular attention to Lev degradation to reduce uncertainties in BB source identification. PM_2.5_ samples were collected from October to December 2021 and analyzed for PM_2.5_ mass, carbonaceous components, water-soluble inorganic ions (WSIs), and anhydrosugars. Lev_no‑chem_ was estimated using the correction factor, and diagnostic ratios were used to classify BB sources. In addition, fire spot mapping and backward trajectory analyses were used to evaluate potential contributions from long-range transport. This study is the first in SEA to incorporate Lev_no‑chem_ into BB source identification, providing new insights into BB emissions in HCMC.

## 2 Results and Discussion

### 2.1 Overall Mass Concentration

#### 2.1.1 PM_2.5_ Mass

During the sampling period, the mass concentration of PM_2.5_ ranged from 12.6 to 46.9 μg m^–3^, with an average (mean ± standard deviation) of 26.6 ± 8.8 μg m^–3^ (Table 1). The PM_2.5_ level in this study aligns with previous observations in HCMC, which reported averages of 28.2 ± 11.2 μg m^–3^ (2016–2019),^35^ 28.4 ± 11.6 μg m^–3^ (2019–2020),^36^ and 21.0 ± 13.9 μg m^–3^ (2021).^39^ In contrast, the average concentrations of PM_2.5_ in Hanoi have been notably higher: 53 ± 17 μg m^–3^ in 2018,^27^ 40.2 ± 26.3 μg m^–3^ in 2019–2020,^31^ and 40.2 ± 15.4 ^29^ and 46 μg m^–3^ ^30^ in 2020. The higher PM_2.5_ concentrations in Hanoi (northern Vietnam) are likely attributed to local emissions from coal and heavy fuel oil combustion, BB, and to long-range transported aerosols from southern China.^27,30,31^ Meanwhile, the PM_2.5_ concentrations in HCMC (southern Vietnam) are dominated by local emissions (e.g., traffic, BB, and cooking) rather than long-range transport.^34,37,40^

**Table 1:** Concentration of PM_2.5_ and Chemical Composition in Different Periods

		Whole period (*n* = 32)	Rainy season (*n* = 23)		Dry season (*n* = 9)		
Variables	Unit	Avg^a^ ± SD^b^	Avg ± SD	Range	Avg ± SD	Range	*p*-value
PM_2.5_	μg m^–3^	26.6 ± 8.8	23.3 ± 6.2	12.6–35.3	35.0 ± 9.3	19.2–46.9	0.005*
OC	μg m^–3^	8.1 ± 3.0	7.3 ± 2.2	3.0–12.4	10.2 ± 3.8	3.7–16.3	0.053
EC	μg m^–3^	2.9 ± 1.0	2.7 ± 0.7	0.9–3.9	3.4 ± 1.3	1.3–6.0	0.163
OC/EC		2.8 ± 0.4	2.8 ± 0.4	2.1–3.5	3.0 ± 0.3	2.7–3.8	0.06
WSOC	μg m^–3^	5.0 ± 1.9	4.2 ± 1.0	2.2–6.0	7.1 ± 2.1	3.6–11.0	0.003*
WSOC/OC		0.63 ± 0.13	0.58 ± 0.09	0.45–0.77	0.73 ± 0.16	0.43–0.97	0.021*
POC	μg m^–3^	6.1 ± 2.0	5.6 ± 1.5	1.9–8.2	7.1 ± 2.8	2.8–12.6	0.163
SOC	μg m^–3^	2.1 ± 1.4	1.7 ± 1.2	0.0–4.8	3.1 ± 1.4	1.0–5.7	0.02*
WSIs	μg m^–3^	6.61 ± 1.86	6.06 ± 1.53	2.98–8.48	8.02 ± 1.97	5.47–11.37	0.02*
Na^+^	μg m^–3^	0.38 ± 0.17	0.33 ± 0.12	0.16–0.63	0.52 ± 0.19	0.27–0.95	0.007*
NH_4_^+^	μg m^–3^	0.68 ± 0.33	0.62 ± 0.32	0.17–1.28	0.83 ± 0.33	0.4–1.49	0.122
K^+^	μg m^–3^	1.05 ± 0.41	0.98 ± 0.37	0.45–1.94	1.22 ± 0.46	0.51–1.9	0.176
Cl^–^	μg m^–3^	0.22 ± 0.12	0.24 ± 0.09	0.08–0.46	0.19 ± 0.17	0.02–0.59	0.094
NO_3_^–^	μg m^–3^	1.19 ± 0.66	0.92 ± 0.3	0.47–1.69	1.88 ± 0.83	1.04–3.36	0.009*
SO_4_^2–^	μg m^–3^	2.91 ± 0.84	2.83 ± 0.90	1.21–4.41	3.11 ± 0.67	2.02–3.86	0.348
C_2_O_4_^2–^	μg m^–3^	0.18 ± 0.08	0.15 ± 0.06	0.0–0.26	0.26 ± 0.04	0.2–0.32	0.001*
Lev	ng m^–3^	210 ± 82	214 ± 83	67–460	201 ± 84	84–320	0.685
Man	ng m^–3^	13 ± 5	12 ± 4	4–23	14 ± 6	6–24	0.554
Gal	ng m^–3^	4 ± 2	3 ± 2	1–8	5 ± 3	3–10	0.086
K_BB_^+^ (nss-K^+^)	μg m^–3^	1.03 ± 0.40	0.97 ± 0.37	0.44–1.93	1.21 ± 0.46	0.49–1.87	0.185
Lev_no‑chem_	ng m^–3^	1766 ± 637	1753 ± 631	621–3281	1798 ± 691	762–2572	0.866

a Average concentration.

b Standard deviation. *p-value < 0.05.

The PM_2.5_ mass concentrations were significantly lower (*p* < 0.05, Table 1) during the rainy season (23.3 ± 6.2 μg m^–3^) compared to the dry season (35.0 ± 9.3 μg m^–3^). The lower PM_2.5_ concentrations observed during the rainy season are attributed to wet deposition associated with high precipitation events (Figure 1a,c), consistent with previous reports.^33,36,41^

**Figure 1:**
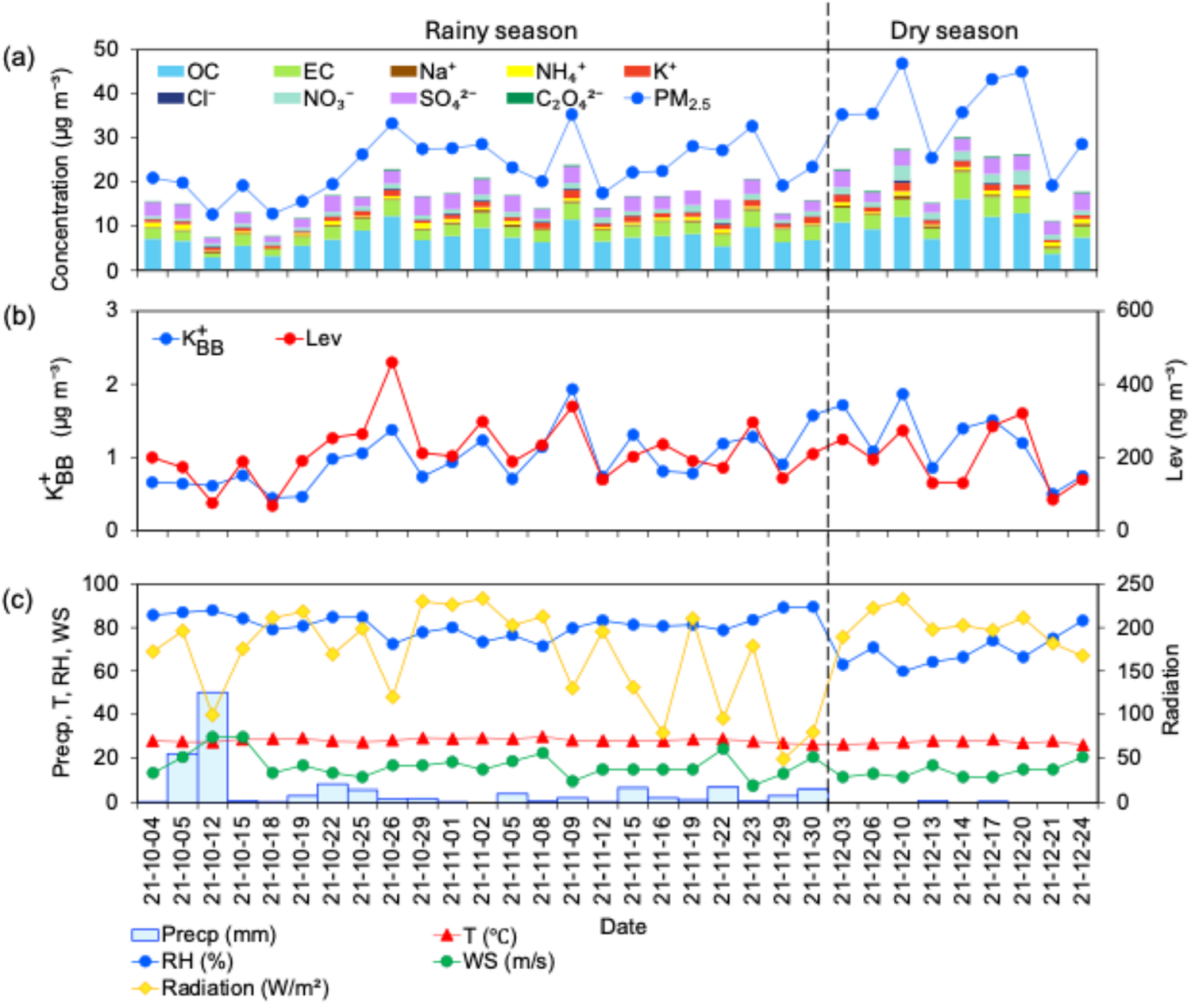
Temporal variation of (a) PM_2.5_ and its chemical composition, (b) K_BB_^+^ and Lev, and (c) meteorological data. The dashed black line divides the sampling period into two seasons: rainy season (left side) and dry season (right side).

#### 2.1.2 WSIs

During the campaign, WSIs accounted for 25.6 ± 4.4% of the total PM_2.5_ mass, averaging 6.61 ± 1.86 μg m^–3^. SO_4_^2–^ was the most abundant ion among the WSIs, contributing 11.4% to the PM_2.5_ mass, followed by NO_3_^–^ (4.4%), K^+^ (4.0%), and NH_4_^+^ (2.6%) (Table 1). Seasonal comparisons revealed that only Na^+^, NO_3_^–^, and C_2_O_4_^2–^ exhibited statistically significant increases during the dry season (*p* < 0.05, Table 1). The long-range transport of fresh sea salt contributed to the higher Na^+^ concentration in the dry season.^37^ Meanwhile, wet deposition was responsible for the lower NO_3_^–^ and C_2_O_4_^2–^ concentrations in the rainy season.^36^ Furthermore, the NO_3_^–^/SO_4_^2–^ mass ratio remained below 1 (0.42 ± 0.20), consistent with our previous finding of 0.38 ± 0.19, indicating substantial emissions from stationary sources.^36^

#### 2.1.3 Carbonaceous Compounds

The average mass concentrations of OC and EC were 8.1 ± 3.0 and 2.9 ± 1.0 μg m^–3^, accounting for 30.7 ± 5.3 and 11.1 ± 2.6% of the PM_2.5_ mass, respectively (Table 1). The OC/EC ratios ranged from 2.1 to 3.8, with an average of 2.8 ± 0.4. No significant seasonal variations were observed in OC and EC, or OC/EC ratios (*p* > 0.05). These values were comparable to those observed during our previous 2019–2020 campaign (OC: 8.8 ± 4.0 μg m^–3^, EC: 2.8 ± 1.5 μg m^–3^, OC/EC: 3.3 ± 0.6), suggesting that primary carbonaceous emissions at this site remained relatively stable.

In contrast, WSOC concentrations and WSOC/OC mass ratios were higher than those observed in the 2019–2020 campaign. During the 2021 campaign, the average WSOC concentration and WSOC/OC ratio were 5.0 ± 1.9 μg m^–3^ and 0.63 ± 0.13, respectively, compared to 3.2 ± 2.0 μg m^–3^ and 0.46 ± 0.10 in 2019–2020.^36^ These elevated WSOC/OC ratios suggest enhanced atmospheric oxidation of organic aerosols during the sampling period. Seasonal differences further indicate enhanced secondary formation during the dry season. The WSOC/OC ratio was statistically higher (*p* < 0.05) in the dry season (0.73 ± 0.16) than in the rainy season (0.58 ± 0.09) (Table 1). Previous studies reported WSOC/OC ratios of ∼0.7 for aged aerosols, ∼0.6 for long-range transported aerosols, ∼0.4 for BB influence, and 0.27 for urban aerosols,^1,6^ indicating that higher ratios generally reflect more oxidized organic aerosols. Consistent with this interpretation, WSOC showed relatively strong correlations with both POC (*r =* 0.74, *p* < 0.01) and SOC (*r =* 0.67, *p* < 0.01) during the rainy season, suggesting mixed contributions from primary emissions and secondary formation. In contrast, during the dry season, the correlation between WSOC and POC was weak (*r =* 0.49), while its correlation with SOC was strong (*r =* 0.94, *p* < 0.01). In addition, SOC concentrations increased significantly (*p* < 0.05) from 1.7 ± 1.2 μg m^–3^ in the rainy season to 3.1 ± 1.4 μg m^–3^ in the dry season.

These results indicate that secondary organic aerosol formation was enhanced during the dry season under strong photochemical conditions. Such atmospheric processing may also promote the chemical degradation of BB tracers such as Lev.

### 2.2 Characteristics of BB Tracers

The average concentration of Lev was 210 ± 82 ng m^–3^, accounting for ∼92.6% of the total anhydrosugar mass and 0.81% of the PM_2.5_ mass, making it the most abundant species among the measured anhydrosugars. Man and Gal were the minor species, with average concentrations of 13 ± 5 ng m^–3^ (5.7% of total anhydrosugar mass) and 4 ± 2 ng m^–3^ (1.8%), respectively. The concentration of Lev in this study was lower than those reported for Hanoi in 2020 (400 ± 400 ng m^–3^, 0.75% of PM_2.5_ mass)^32^ and in 2019–2020 (380 ± 350 ng m^–3^, 1.65% of PM_2.5_ mass),^30^ as well as for Sonla in 2013 (1620 ± 890 ng m^–3^, 2.23 ± 0.05% of PM_2.5_ mass).^45^

Although Lev, Man, and Gal concentrations exhibited no seasonal variations (*p* > 0.05, Table 1), their correlation patterns with other species varied between seasons. During the rainy season, Lev and Man exhibited a significant correlation with each other (*r =* 0.92, *p* < 0.01) and moderate to strong correlations with OC, EC, and POC (*r >* 0.82, *p* < 0.01) (Figure 2a), indicating their BB source was related to primary emissions. In contrast, during the dry season, Lev, Man, and Gal showed moderate to significant correlations with each other (*r >* 0.71, *p* < 0.05), with WSOC (*r >* 0.87, *p* < 0.01), and with SOC (*r >* 0.77, *p* < 0.01) (Figure 2b), suggesting that they originate from related sources and have a strong association with secondary organic aerosol formation. These findings imply that PM_2.5_ in HCMC was substantially influenced by BB emissions during the sampling period, with the dry season showing clearer evidence of secondary aerosol formation from BB-derived precursors.

**Figure 2:**
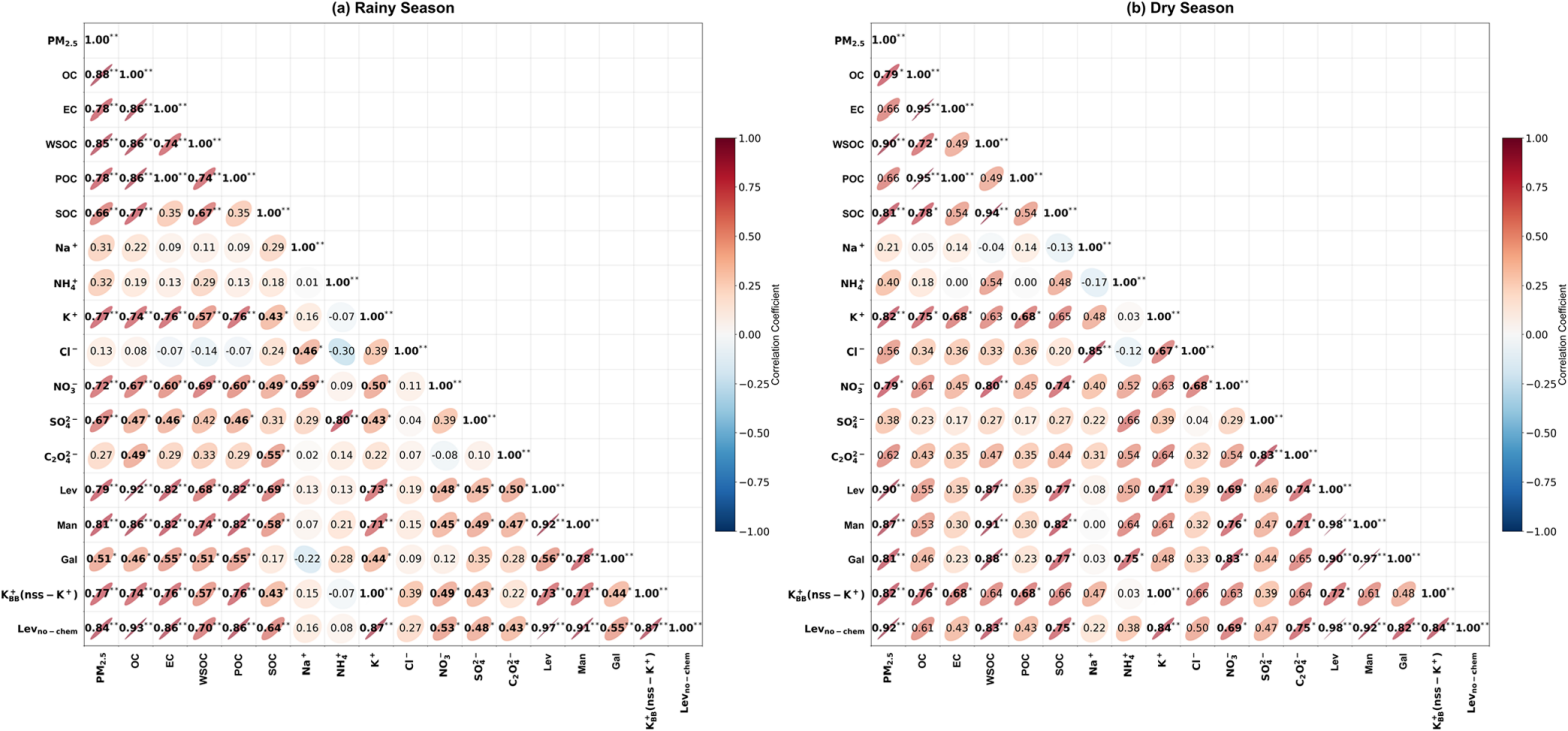
Correlations among PM_2.5_ and its chemical components in the rainy season (a) and the dry season (b).

However, Gal showed a weak correlation with Lev (*r =* 0.56, *p* < 0.01) and a good correlation with Man (*r =* 0.78, *p* < 0.01) during the rainy season. The complex correlations between Gal to Lev and Man during the dry and rainy seasons can be explained by their different degradation rates. Wang et al.^13^ reported that the degradation rates of Man and Gal were 34 and 40% higher than that of Lev, respectively, due to differences in their chemical steric structures, especially the C_3–5_ skeletons and OH groups. The larger steric hindrance at the C_4_-position of Lev, compared to Man and Gal, makes Lev more chemically stable in the environment and thus less soluble than Man and Gal.^12^ Therefore, we hypothesize that the weak and moderate correlations of Gal with Lev and Man during the rainy season stem from the low concentration of Gal in the atmosphere (Table 1) and its higher degradation rate compared to Man and Lev. Further studies are required to substantiate this conjecture.

The average concentration of K_BB_^+^ (also known as non-sea-salt potassium, nss-K^+^) was 1.03 ± 0.40 μg m^–3^ during the sampling period. The K_BB_^+^/K^+^ (also known as nss-K^+^/K^+^) mass ratio can be used to estimate the relative contribution of BB, where a low ratio (<0.1) indicates the influence of air masses from the ocean and a high ratio (>0.1) represents the influence of BB.^46,47^ In this study, the K_BB_^+^/K^+^ ratio was 0.99, indicating that BB was the predominant source of K^+^ in PM_2.5_ during the sampling period. Additionally, K_BB_^+^ showed good correlations with Lev in both the rainy season (*r =* 0.73, *p* < 0.01) and the dry season (*r =* 0.72, *p* < 0.05) (Figure 2). The corresponding temporal variations of K_BB_^+^ and Lev were also observed during the sampling period (Figure 1b). Hence, these results indicate that K_BB_^+^ and Lev originated from a similar type of BB emission during the sampling period.

### 2.3 Source Identification of BB Based on Diagnostic Ratios Considering Levoglucosan Degradation

#### 2.3.1 Estimation of Levoglucosan Degradation

The correction factor *x* ranged from 0.10 to 0.15, with an average of 0.12 ± 0.01 (Table 2), indicating that approximately 88% of the initial Lev in HCMC had already degraded prior to arrival at the sampling site. Consequently, the average concentration of Lev_no‑chem_ was estimated to be 1766 ± 637 ng m^–3^, which is eight times greater than the measured value of Lev in PM_2.5_ samples (210 ± 82 ng m^–3^). These results indicate considerable degradation of Lev in the atmosphere, causing uncertainties in identifying and estimating the impact of BB on the PM_2.5_ level at the receptor site. The *x* value in HCMC was lower than those reported for Changchun (0.14 ± 0.04),^22^ Changzhou (0.13 ± 0.07),^21^ and Pu’er (0.18 ± 0.05)^48^ in China (Table 2), suggesting that Lev in HCMC experienced strong degradation compared to these cities.

**Table 2:** Comparison of Levoglucosan in PM_2.5_ Considering the Levoglucosan Degradation in This Study with Those Reported in the Literature^a^

Sampling site	HCMC, Vietnam	Chang-chun, China	Changzhou, China	Yunnan, China
Reference	This study	Hong et al.,^22^	Li et al.,^21^	Shi et al.,^48^
Site type	Urban	Urban	Urban	Rural
Sampling time	Oct–Dec 2021	May 2017–May 2018	Nov 2020–Mar 2021	Mar–May 2022
**Species (unit)**				
K^+^ (μg m^–3^)	1.05 ± 0.41	0.57 ± 0.55	0.41 ± 0.61	1.12 ± 0.64
K_BB_^+^ (μg m^–3^)	1.03 ± 0.4	0.55 ± 0.55	0.39 ± 0.59	0.98 ± 0.64
Lev (ng m^–3^)	210 ± 82	211 ± 293	54.2 ± 80.2	431 ± 171
Lev_no‑chem_ (ng m^–3^)	1766 ± 637	1250 ± 1472	418.3 ± 422.2	2592 ± 1339
Man (ng m^–3^)	13 ± 5	19 ± 20	4.3 ± 5.6	22.7 ± 6.3
*x*	0.12 ± 0.01	0.14 ± 0.04	0.13 ± 0.07	0.18 ± 0.05
Lev/Man	16.7 ± 2.6	11.1^b^	14.1 ± 6.6	18.3 ± 3.8
Lev/K_BB_^+^	0.21 ± 0.07	0.3 ± 0.2	0.22 ± 0.25	0.56 ± 0.28
Lev_no‑chem_/K_BB_^+^	1.76 ± 0.39	2.1 ± 1.0	1.46 ± 0.86	2.94 ± 0.63

a Notes: The data represents mean ± standard deviation. “*x*” indicates the degradation rate of Levoglucosan.

b data was inferred from mean/mean.

Regarding seasonal variations, the value of *x* showed a significant difference (Welch’s *t* test, *p* < 0.05) between the rainy season (0.12 ± 0.01) and the dry season (0.11 ± 0.01), implying that Lev was more degraded during the dry season than in the rainy season. Meteorological factors such as high temperatures (25–35 °C), high RH (>85%), and intense solar radiation contribute to faster Lev degradation.^12,15,20^ During the 2021 campaign, the average temperatures were 28.1 ± 0.8 °C (rainy) and 27.1 ± 0.8 °C (dry), while the average RH values were 81.4 ± 5.0 and 69.2 ± 7.2%, respectively. Furthermore, Lev can be oxidized by gaseous oxidants such as ozone (O_3_), OH radicals, and NO_3_ radicals in the aerosol phase.^10,15,18^ Although OH and NO_3_ radicals were not measured in this study, their generation is associated with the presence of O_3_ and NO_2_ gases in the atmosphere.^49,50^ Ho et al.^51^ reported the highest concentration of O_3_ in December 2017, highlighting elevated levels of O_3_ and NO_2_ in the final months of the year, especially in the low rainfall period (November–December) in HCMC. During the dry season, Lev_no‑chem_ exhibited significant correlations with WSOC (*r =* 0.83, *p* < 0.01) and SOC (*r =* 0.75, *p* < 0.05) (Figure 2), indicating a strong association between enhanced photochemical conditions and BB pollutants. Therefore, our results suggest that Lev degradation in the rainy season is influenced by high RH and temperatures. Meanwhile, the enhanced degradation rate of Lev in the dry season is driven by both meteorological factors such as high temperature and intense solar radiation (Figure 1c) and higher levels of O_3_ and NO_2_. However, further studies on the seasonal variation of Lev degradation in HCMC are needed for a better understanding.

#### 2.3.2 Source Identification Based on Diagnostic Ratios

The mass ratios of Lev/Man, Lev/K^+^, and OC/Lev in PM_2.5_ from fresh BB emissions are used to classify the fuel type of BB.^13,14,52,53^ Figure 3 was adapted from Cheng et al.^13^ for various BB emissions based on the Lev/Man and Lev/K^+^ ratios of U.S. hardwood and softwood,^54–60^ U.S. needles, duff, and grass,^53^ and Asian rice straw.^52,61^ Furthermore, Sang et al.^14^ summarized and reported average OC/Lev ratios in PM_2.5_ of 15.1 ± 4.3 for crop residue, 11.4 ± 5.8 for softwood, and 6.9 ± 4.0 for hardwood.

**Figure 3:**
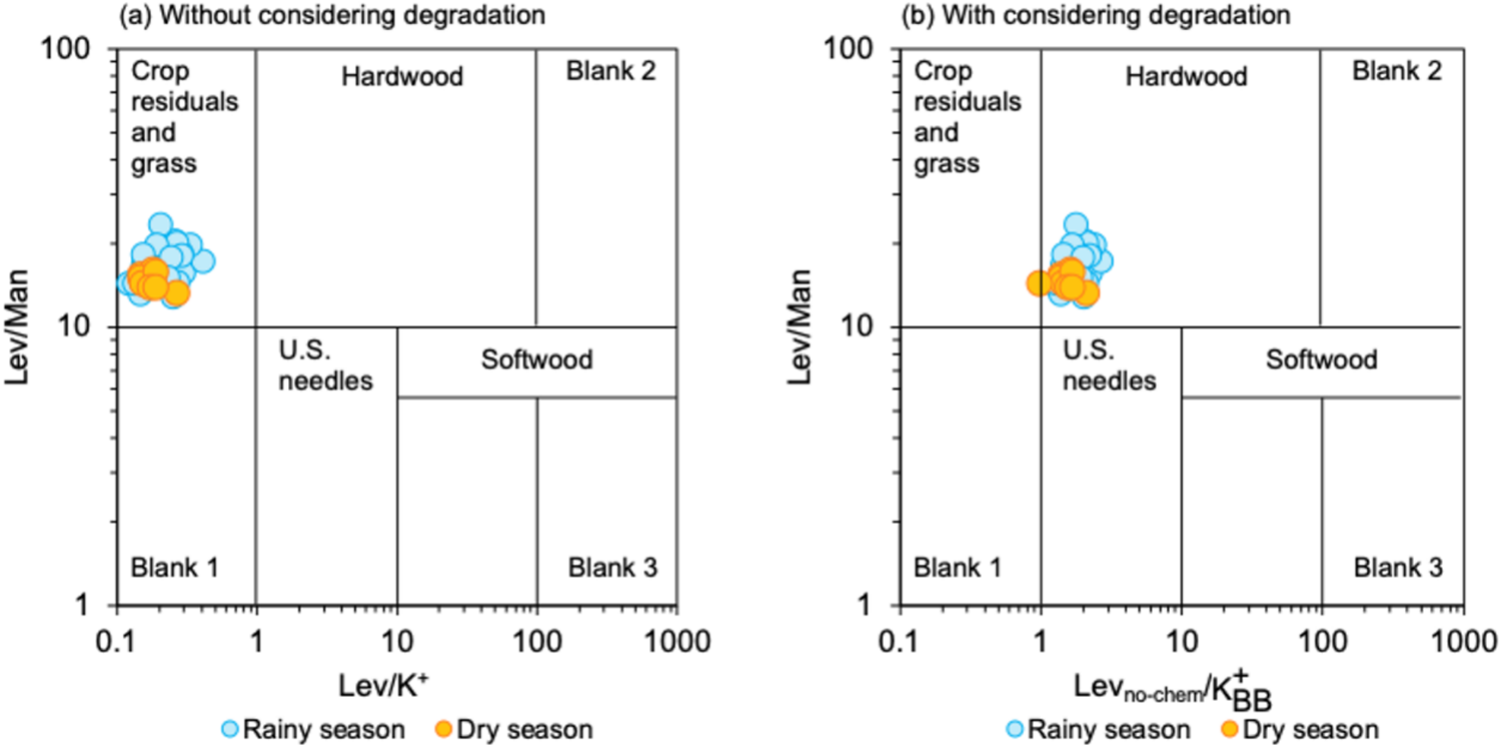
Representative ranges of Lev/K^+^ (*x*-axis) and Lev/Man (*y*-axis) ratios for different types of BB fuel. Results from Lev in PM_2.5_ samples collected in this study are also shown for comparison with two cases: (a) without considering the degradation of Lev and (b) with considering the degradation of Lev (Lev_no‑chem_). Figure adapted from Cheng et al.,^13^ licensed under Creative Commons Attribution 3.0.

In this study, without considering Lev degradation, the average ratio and range of Lev/Man was 16.7 ± 2.6 (12.9–23.6) and Lev/K_BB_^+^ was 0.21 ± 0.07 (0.09–0.41) (Table 2), suggesting potential origins from crop residue and grass burning (Figure 3a). However, the OC/Lev ratio was 38.5 ± 7.6 (26.9–54.8), which is substantially higher than those reported by Sang et al.^14^ as mentioned above, suggesting an unidentified source. Therefore, using Lev/Man, Lev/K_BB_^+^, and OC/Lev ratios without accounting for Lev degradation suggests inconsistent BB source profiles and reduces accuracy in BB source identification in HCMC.

In considering Lev degradation, the average ratio of Lev_no‑chem_/K_BB_^+^ was 1.76 ± 0.39 (0.97–2.67) (Table 2), indicating emissions from hardwood combustion (Figure 3b). Regarding the Lev/Man ratio, although Wang et al.^12^ reported a higher degradation rate of Man compared to Lev, we assumed equivalent degradation rates for both species in this study. Consequently, the calculated Lev/Man ratios reported in this study may underestimate the initial Lev_no‑chem_/Man_no‑chem_ ratios, highlighting the need for further investigation into the chemical degradation of all anhydrosugars. However, previous studies have shown that Lev/Man ratios greater than 10 indicate emissions from hardwood burning.^13,61^ Therefore, regardless of this potential underestimation, the Lev/Man ratios observed in HCMC consistently remain above the threshold, indicating hardwood combustion as the dominant emission source (Figure 3b). Furthermore, the Lev/Man ratios and Lev_no‑chem_/K_BB_^+^ ratios were also comparable to those produced by metal-grill charcoal combustion, at 13.14 ± 3.86 and 1.36 ± 1.16, respectively.^62^ In terms of OC and Lev_no‑chem_, the OC/Lev_no‑chem_ ratio was 4.54 ± 0.74, which lies within the range of OC/Lev for hardwood burning (6.9 ± 4.0).^14^ Hence, by accounting for the degradation of Lev, these diagnostic ratios of Lev_no‑chem_/K_BB_^+^, Lev/Man, and OC/Lev_no‑chem_ indicate consistent results, suggesting that hardwood and/or hardwood charcoal were the primary types of BB emissions in HCMC during the sampling period.

#### 2.3.3 Geological Origins of BB Emission

To identify the geographical origins of BB emissions and their long-range transport effects, we conducted three geographic information system (GIS) analyses: fire spot mapping at a high confidence level (80–100%) (Figure 4); backward trajectory cluster analysis (Figure 5); and weighted concentration weighted trajectory (WCWT) analysis (Figure 6). Figure 4 demonstrates that fire spots were primarily concentrated 20–50 km west and scattered toward the north/northeast of the sampling site. Previous studies have suggested that the long-range transport of emissions from crop open burning in the Mekong Delta influences air quality in HCMC in May^38^ and September.^33^ This study was conducted from October to December, which coincided with the harvest period of the autumn–winter rice crop in southern Vietnam (Figure S1). According to the Foreign Agricultural Service,^63^ rice crops were primarily distributed to the south/southwest of HCMC (Figure S2), suggesting a potential influence from crop open burning.

**Figure 4:**
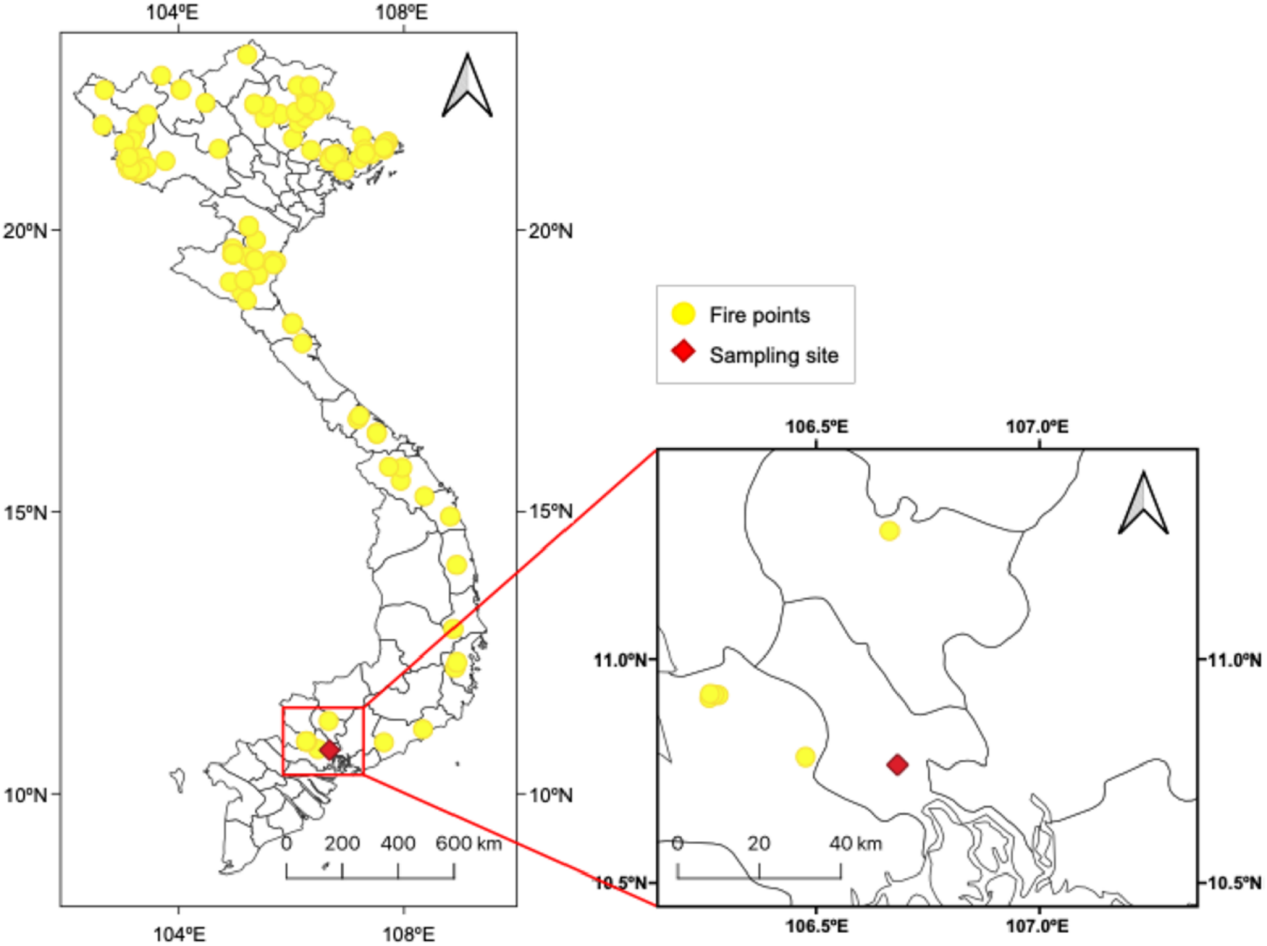
Distribution of fire spots with high confidence (80–100%). Note: This map is an image of the country, not its actual border.

**Figure 5:**
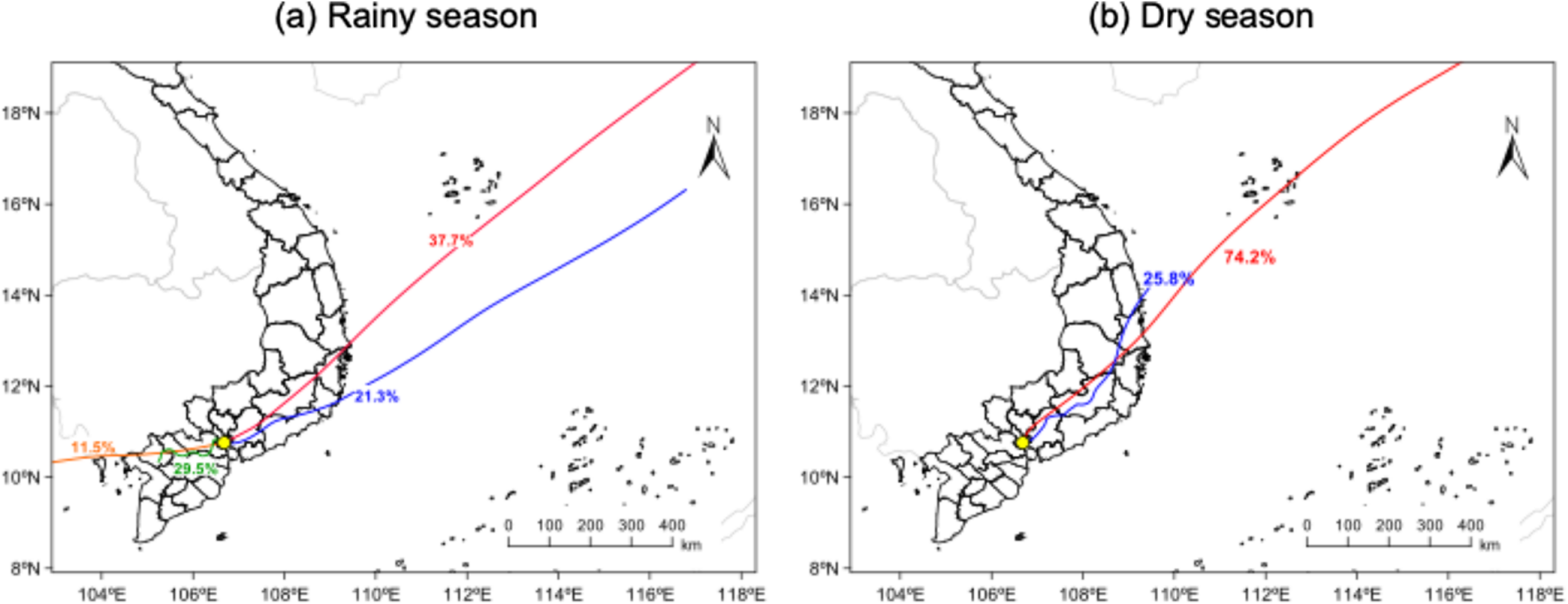
Analytical results of the 72-h air mass backward trajectories at 500 m in the rainy season (a) and the dry season (b). The yellow dot represents the sampling site. Note: This map is an image of the country, not its actual border.

**Figure 6:**
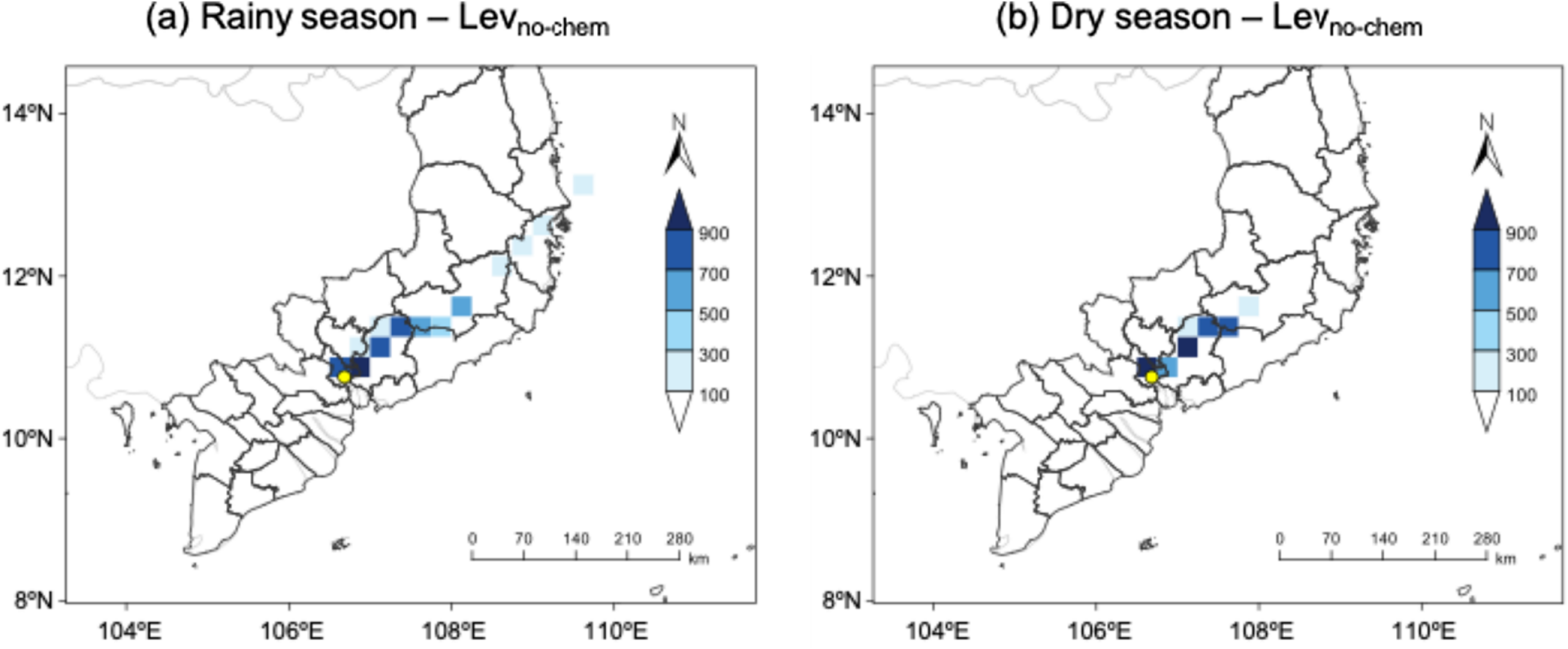
WCWT analysis of Lev_no‑chem_ in the rainy season (a) and dry season (b). The yellow dot represents the sampling site. The unit of WCWT value: ng m^–3^. Note: This map is an image of the country, not its actual border.

However, the cluster analysis of the 72-h air mass backward trajectories and the WCWT analyses imply different results. Although two clusters (∼40%) originated from the west during the rainy season (Figure 5), the WCWT analysis indicates that the potential emission regions of Lev_no‑chem_ were from local areas and from the north/northeast of the sampling site (Figure 6). Similar spatial patterns were also observed for Lev during the sampling period, as well as for PM_2.5_, OC, and K_BB_^+^ (Figure S3). The WCWT analyses in this study were consistent with those in previous studies in HCMC,^36–38,64^ confirming that the observed aerosols primarily originated from local areas and were partially transported from the north and northeast of HCMC during the campaign.

The lack of BB emissions from western trajectories may be explained by the limited crop residue burning in the Mekong Delta during the sampling period. A study in Long An province, a fire hotspot area located just west of HCMC (Figure 4), reported that only ∼28% of rice straw was burned during the autumn–winter crop season, contributing ∼20% of total PM_2.5_ emissions in 2021–2022 period.^65^ Dung and Thy^66^ also reported that only ∼50% of rice straw was burned across the Mekong Delta region during the autumn–winter crop season in 2020, leading to a substantial reductions in gaseous pollutants (e.g., CH_4_, NO_*x*_, and CO) from agricultural waste burning emissions compared to 2012 levels. Furthermore, the Vietnamese government has implemented regulations to mitigate crop open burning activities (Law No. 72/2020/QH14 on Environmental Protection) and officially prohibited these practices in 2022 (Decree 45/2022/NĐ-CP). Hence, these policy actions and previous findings further strengthen our hypothesis that the influence of crop residue burning on air quality in HCMC during the study period was negligible.

Furthermore, previous studies have indicated the use of hardwood and hardwood charcoal as dominant cooking fuels in southern Vietnam, strengthening our findings based on diagnostic ratios of Lev_no‑chem_ and WCWT results. Toan et al.^67^ reported that charcoal produced in southern Vietnam is mainly made from *Eucalyptus* and *Rhizophora* species, both of which belong to the hardwood category. Similarly, Tho et al.^68^ indicated that more than 80% of the charcoal produced in Vietnam is derived from regenerated plantation timber, including its salvaged and waste wood, primarily made from hardwood species such as *Eucalyptus*, *Acacia*, and *Aquilaria*. In HCMC, numerous street vendors and restaurants conduct outdoor cooking and frequently use charcoal as a fuel source. Kim Oanh et al.^69^ also observed significant PM_2.5_ emissions from charcoal combustion (mangrove and/or lump charcoal) during outdoor cooking activities in HCMC, while Huy et al.^70^ estimated that more than 99% of PM_2.5_ emissions from residential combustion activities originated from biofuels (primarily charcoal). According to Chi et al.^34^ and Dat et al.,^40^ significant polycyclic aromatic hydrocarbon emissions in HCMC’s PM_2.5_ arise from local vendor cooking and general wood/coal combustion, respectively. Taken together, these lines of evidence reinforce our conclusion that BB emissions in HCMC primarily originate from hardwood and/or hardwood charcoal used for local and commercial cooking, with major contributions from local urban areas and partial transport from the northern and northeastern regions.

### 2.4 Limitations of this Study

The primary limitation of this study is the relatively small sample size (N = 32 samples), particularly during the dry season (N = 9), which may limit the long-term representativeness of the seasonal comparisons. In SEA, the transition from the rainy to the dry season often coincides with increased BB activities. While our results show significant seasonal differences in certain parameters (Table 1), we acknowledge that a 3-month window might not capture the full intensity of the BB season.

To mitigate statistical bias and ensure the reliability of our comparisons, we utilized Welch’s *t* test, which is robust for comparing means with unequal variances and sample sizes.^71–73^ Despite the limited sample size, several parameters (e.g., PM_2.5_ mass, OC, and EC concentrations) are highly consistent with our previous long-term observations in HCMC.^36,37^ Therefore, this study serves as a preliminary study providing new insights into cooking emissions in Vietnam. However, a comprehensive year-round study at multiple locations (urban, suburban, and background) remains necessary to fully characterize the regional air quality and the peak impact of BB sources.

## 3 Conclusions

This study represents the first investigation in Vietnam and SEA to incorporate Lev degradation into BB source identification. Analysis of PM_2.5_ samples collected in HCMC revealed a notably high Lev degradation rate of approximately 88%, driven by the region’s tropical climate with high temperatures, humidity, intense solar radiation, and elevated atmospheric oxidants (e.g., O_3_ and NO_2_).

Accounting for Lev degradation was critical: without correction, diagnostic ratios (Lev/Man, Lev/K_BB_^+^, and OC/Lev) incorrectly suggested crop residue and grass burning as dominant BB sources. After correction, the ratios (Lev/Man, Lev_no‑chem_/K_BB_^+^, and OC/Lev_no‑chem_) consistently indicated that hardwood and hardwood charcoal combustion are the actual predominant BB sources.

Long-range transport effects were assessed using fire spot maps, WCWT analysis, and regional data on polycyclic aromatic hydrocarbon emissions, charcoal production, and crop production. These analyses confirm that BB in HCMC primarily originates from local and commercial cooking activities using hardwood and/or hardwood charcoal, with minor contributions from the north and northeast.

Overall, our results highlight the necessity of using Lev_no‑chem_ for accurate BB source identification in SEA, where humid tropical environments substantially shorten pollutant lifetimes, and demonstrate that local cooking emissions are a major BB contributor to PM_2.5_ in HCMC.

## 4 Materials and Methods

### 4.1 Aerosol Sampling

Details of the sampling location and sampling method are provided in our previous studies.^36,37^ In brief, samples were collected on the rooftop (49 m above ground level) of a 12-story building at the Vietnam National University Ho Chi Minh City – University of Science (10°45′43.6″ N; 106°40′52.8″ E). The sampling site is in central HCMC, a densely populated area with heavy traffic and residential activities, surrounded by industrial zones within a 20–50 km radius and situated 36–200 km northeast of the Mekong Delta.

A total of 32 PM_2.5_ samples were collected between October and December 2021, including 23 samples during the rainy season (October–November) and 9 samples during the dry season (December). A set of two IMPACT samplers (SKC, Inc., 10 L/min) simultaneously collected PM_2.5_ on 47 mm PTFE and quartz fiber filters over a 24-h period. PTFE and quartz fiber filters were stored in the refrigerator (4 °C) and freezer (−20 °C), respectively, until chemical analyses.

### 4.2 Measurement of PM_2.5_ Mass and Its Chemical Composition

Analytical procedures for PM_2.5_ mass, WSIs (Cl^–^, NO_3_^–^, SO_4_^2–^, C_2_O_4_^2–^, Na^+^, NH_4_^+^, and K^+^), and carbonaceous components (organic carbon–OC, elemental carbon–EC, and water-soluble organic carbon–WSOC) followed the protocols described in our previous study.^36^ Briefly, PTFE filters were used to quantify PM_2.5_ mass with a microbalance (XSE 205DU, Mettler Toledo) and WSIs with ion chromatography (Met-831, Metrohm). Meanwhile, quartz fiber filters were used to determine OC and EC with a carbon analyzer (Lab OC-EC Aerosol Analyzer, Sunset Laboratory) and WSOC with a total organic carbon analyzer (TOC-L, Shimadzu). Estimation of primary organic carbon (POC) and secondary organic carbon (SOC) based on the primary OC/EC ratio was also conducted as described in Tran et al.^36^

Anhydrosugars (Lev, Man, and Gal) in PM_2.5_ were quantified using gas chromatography–mass spectrometry (GC-MS) following the protocol of Fujii et al.,^74,75^ which included the extraction method, GC program, and MS settings. Aliquots (area: 1.01 cm^2^) of the quartz fiber filters were spiked with internal standards of Lev-^13^ *C*_6_, palmitic acid-*d*_31_, and cholesterol-*d*_7_, and extracted by ultrasonic agitation for 30 min in 500 μL of a dichloromethane/methanol mixture (3/1, *v*/*v*). Extracts were filtered through PTFE syringe filters (0.2 μm, Mini-UniPrep G2, Whatman). Subsequently, 100 μL of the extracts were dried completely under a gentle nitrogen stream and derivatized with 50 μL of *N,O*-bis-(trimethylsilyl)-trifluoroacetamide containing 1% trimethylchlorosilane and 10 μL of pyridine. The mixtures were incubated at 70 °C for 3 h, diluted with 140 μL of *n*-hexane, and subsequently injected into the GC-MS.

The anhydrosugars were analyzed using the Shimadzu GC-MS system (Shimadzu GCMS-QP2020) equipped with a 30 m HP-5MS column (film thickness: 0.25 μm; inner diameter: 0.25 mm). High-purity helium (99.99995%) served as the carrier gas at a constant flow rate of 1.0 mL min^–1^. Derivatized samples (1.0 μL) were injected into the GC in splitless mode at an injector temperature of 280 °C. The temperature of the GC oven was programmed as follows: isothermal at 80 °C for 1.5 min, 80–150 °C at 8 °C min^–1^, 150–190 °C at 3 °C min^–1^, 190–310 °C at 15 °C min^–1^, and then 310 °C for 5 min. The data of quantitative analysis were obtained in the electron impact mode of 70 eV. The MS was conducted under the selected ion monitoring mode, and the monitored ions for the quantification of Lev, Man, and Gal were 217. For the internal standards, the monitored ions of Lev-^13^ *C*_6_, palmitic acid-*d*_31_, and cholesterol-*d*_7_ were 220, 344, and 336, respectively. Blank corrections using laboratory blank filters were not applied, as none of the anhydrosugars were detected in the blank samples.

### 4.3 Estimation of the Levoglucosan Degradation

Levoglucosan has been mentioned as a specific tracer of BB. However, recent studies have observed the considerable degradation rate of Lev, indicating potential uncertainties in estimating the contributions of BB to Lev, OC, and PM_2.5_ concentrations at the receptor site.^19,21,22,48^ To overcome this challenge, Li et al.^19^ developed a correction factor, *x*, using the GEOS-Chem model to estimate the concentration of Lev without chemical degradation (Lev_no‑chem_) as follows:

1 $${\mathrm{Lev}}_{\mathrm{no}‐\mathrm{chem}}=\frac{{\left(\mathrm{Lev}\right)}_{\mathrm{ambient}}}{x}$$

2 $$x=0.18\times \frac{{\left(\mathrm{Lev}\right)}_{\mathrm{ambient}}}{{\left(K_{\mathrm{BB}}^{+}\right)}_{\mathrm{ambient}}}+0.08$$

where *x* represents the freshness of particulate Lev at the sampling site, (Lev)_ambient_ is the measured concentration of Lev in PM_2.5_ samples, and K_BB_^+^ is the concentration of potassium emitted from BB.^19^ The values of 0.18 and 0.08 are the slope and intercept of the modeled Lev/Lev_no‑chem_, which were inferred from the significant correlation (*r =* 0.84) between Lev and K_BB_^+^ (Lev/K_BB_^+^) in 56 global sites (including 24 urban sites, 13 rural sites, 5 forest sites, 8 marine sites, and 6 polar sites).^19^ The value of *x* ranges from 0 for completely degraded air masses to 1 for air masses from fresh BB emissions.^19^ The concentration of K_BB_^+^ is estimated by subtracting the amount emitted from sea salt as shown in eqs 3–7.^21,76^ In this study, the measured concentrations of Ca^2+^ were below the limit of detection (0.02 μg m^–3^), which led to negative values for K_Dust_^+^ and consequently an underestimation of K_BB_^+^. To address this, we excluded K_Dust_^+^ and assumed K_BB_^+^ = nss-K^+^.

3 $$K_{\mathrm{BB}}^{+}=\mathrm{nss}‐K^{+}-K_{\mathrm{Dust}}^{+}$$

4 $$\mathrm{nss}‐K^{+}=K_{\mathrm{aerosol}}^{+}-0.036\times {\mathrm{Na}}_{\mathrm{aerosol}}^{+}$$

5 $$K_{\mathrm{Dust}}^{+}=0.04\times \mathrm{nss}‐{\mathrm{Ca}}^{2+}-{\mathrm{Ca}}_{\mathrm{BB}}^{2+}$$

6 $${\mathrm{Ca}}_{\mathrm{BB}}^{2+}=\mathrm{nss}‐K^{+}/53.96$$

7 $$\mathrm{nss}‐{\mathrm{Ca}}^{2+}={\mathrm{Ca}}_{\mathrm{aerosol}}^{2+}-0.038\times {\mathrm{Na}}_{\mathrm{aerosol}}^{+}$$

### 4.4 Potential Source Contribution Origins

Previous studies indicated that the long-range transport effect of crop open burning in the Mekong Delta potentially influences PM_2.5_ levels in HCMC; therefore, observing the transboundary effects of pollutants is essential.^33,38^ To identify the spatial origins of these transboundary emissions, we performed WCWT analysis using TrajStat software.^77^ By integrating backward air mass trajectories with concentrations of PM_2.5_ mass and its chemical composition, this model estimates which specific grid cells have high potential contributions to pollutants levels at the sampling site. Air mass data were obtained from the 1° × 1° Global Data Assimilation System (GDAS one-degree archive, https://www.ready.noaa.gov/archives.php) provided by the National Oceanic and Atmospheric Administration (NOAA). The coordinates of the starting point were set to the sampling site. We calculated 72-h backward trajectories of air masses within the planetary boundary layer of 500 m. This 72-h duration was chosen because it represents the average atmospheric lifetime of Lev^15,78^ and the typical period to evaluate long-range transport effects of PM_2.5_.^79^ The world map was provided in TrajStat in MeteoInfo.^77^ The shapefile for the administrative boundaries of Vietnam (data set: vnm_admbnda_adm1_gov_20201027) was obtained from the United Nations Office for the Coordination of Humanitarian Affairs (OCHA) via the Humanitarian Data Exchange (https://data.humdata.org/dataset/cod-ab-vnm), and added as a layer using TrajStat in MeteoInfo. Details of the cluster analysis and WCWT analysis were described in our previous studies.^36,37^

The data of fire spots during the sampling period were acquired from the Terra (MOD14A1) and Aqua (MYD14A1) Moderate Resolution Imaging Spectroradiometer (MODIS) fire products, which were developed by NASA and can be accessed through the Fire Information for Resource Management System (FIRMS, https://firms.modaps.eosdis.nasa.gov). The MOD14A1 and MYD14A1 are daily Level 3 fire products that observe fire pixels at a 1-km grid resolution over 24 h. The data set of fire spots is categorized into three classes based on their detection confidence: low (0–30%), nominal (30–80%), and high (80–100%) confidence.^80^ In this study, we only use data with high confidence and exclude data with low and nominal confidence to avoid drawing false conclusions about small fires.^81,82^

Meteorological data (abbreviation, unit), including temperature (*T*, °C), relative humidity (RH, %), precipitation (Precp, mm), and solar radiation (W/m^2^), were obtained from Visual Crossing (www.visualcrossing.com).

### 4.5 Statistical Analyses

To evaluate seasonal variations of PM_2.5_ and its chemical components between the rainy (N = 23) and dry (N = 9) seasons, we primarily employed Welch’s *t* test for statistical comparisons. Unlike the standard Student’s *t* test, Welch’s *t* test is more appropriate here as it accounts for unequal variances and sample sizes.^71–73^

Data normality was first assessed using the Shapiro-Wilk test.^73^ Results showed that most parameters were normally distributed and thus analyzed using the unpaired Welch’s *t* test, while Na^+^ and Cl^–^ exhibited a non-normal distribution (Table S1). Consequently, the nonparametric Mann–Whitney U test was used for these two ions. For all statistical comparisons, the null hypotheses assumed no significant difference between the two seasons. All analyses were performed using Python (version 3.12.2) with the Pandas, NumPy, and SciPy libraries.^83–85^ Statistical significance was established at a 95% confidence level (*p* < 0.05).

## Supplementary Material



## Notes

Additional information for Shapio-Wilk test results and BB source identification, including agricultural information, and WCWT analysis for other chemical composition (PDF)
