# Supplementary material for: Hardwood Burning as a Dominant Source of Fine Particulate Matter from Biomass Burning in Ho Chi Minh City, Vietnam
Source: ACS Omega. 2026 Mar 31;11(14):22198–209. doi: 10.1021/acsomega.5c13491 (PMC13084443; doi:10.1021/acsomega.5c13491)
Supplement: Supplementary file 1 [file ao5c13491_si_001.pdf]

Supporting Information:

Hardwood Burning as a Dominant Source of Fine Particulate Matter from Biomass Burning in Ho Chi Minh City, Vietnam

Ngoc Tran[1,2], Yusuke Fujii[1*], To Thi Hien[3,4], Norimichi Takenaka[1]*

[1] Division of Sustainable System Sciences, Graduate School of Sustainable System Sciences, Osaka Metropolitan University, Sakai 599-8531, Osaka, Japan

[2] Faculty of Environment, Ho Chi Minh City University of Natural Resources and Environment, Ho Chi Minh City 700000, Vietnam

[3] Faculty of Environment, University of Science, Ho Chi Minh City 700000, Vietnam

[4] Vietnam National University, Ho Chi Minh City 700000, Vietnam

*Corresponding authors:

Ngoc Tran: k22402v@omu.ac.jp, Yusuke Fujii: fujii.yusuke@omu.ac.jp

[2] Current address of Ngoc Tran

List of contents

Table S1. Shapiro-Wilk test results for the normal distribution of $PM_{2.5}$ mass and chemical composition concentrations. Note: the null hypothesis assumes that the data are normally distributed.

Figure S1. Crop calendars in Vietnam provided by USDA.

Figure S2. Distribution of autumn rice production provided by USDA. Note: This map is an image of country, not the actual border of land.

Figure S3. WCWT analyses of $PM_{2.5}$ (a–b), OC (c–d), K^{+}_{BB} (e–f), and Lev (g–h) in the rainy season (left) and dry season (right). Blue bars indicate the unit of $PM_{2.5}$, OC, and K^{+}_{BB} in μg m^{-3} and Lev in ng m^{-3}. Yellow dot represents the sampling site. Note: This map is an image of country, not the actual border of land.

Table S1. Shapiro-Wilk test results for the normal distribution of $PM_{2.5}$ mass and chemical composition concentrations. Note: the null hypothesis assumes that the data are normally distributed.

	Rainy season		Dry season	
**Variables**	***p-value***	**Normal distribution**	***p-value***	**Normal distribution**
$PM_{2.5}$	0.72	Yes	0.65	Yes
OC	0.36	Yes	0.96	Yes
EC	0.27	Yes	0.93	Yes
OC/EC	0.26	Yes	0.21	Yes
WSOC	0.64	Yes	0.65	Yes
WSOC/OC	0.38	Yes	0.80	Yes
POC	0.27	Yes	0.93	Yes
SOC	0.25	Yes	0.91	Yes
SOC/OC	0.17	Yes	0.57	Yes
WSIs	0.33	Yes	0.78	Yes
Na^+	0.02	No (Skewed)	0.08	Yes
NH_4^+	0.26	Yes	0.62	Yes
K^+	0.23	Yes	0.97	Yes
Cl^-	0.92	Yes	0.02	No (Skewed)
NO_3^-	0.39	Yes	0.11	Yes
SO_4^{2-}	0.35	Yes	0.33	Yes
$C_2O_4^{2-}$	0.06	Yes	0.87	Yes
Lev	0.07	Yes	0.42	Yes
Man	0.42	Yes	0.50	Yes
Gal	0.25	Yes	0.08	Yes
K_{BB}^+(nss-K^+)	0.22	Yes	0.96	Yes
$Lev_{no\text{-}chem}$	0.32	Yes	0.14	Yes

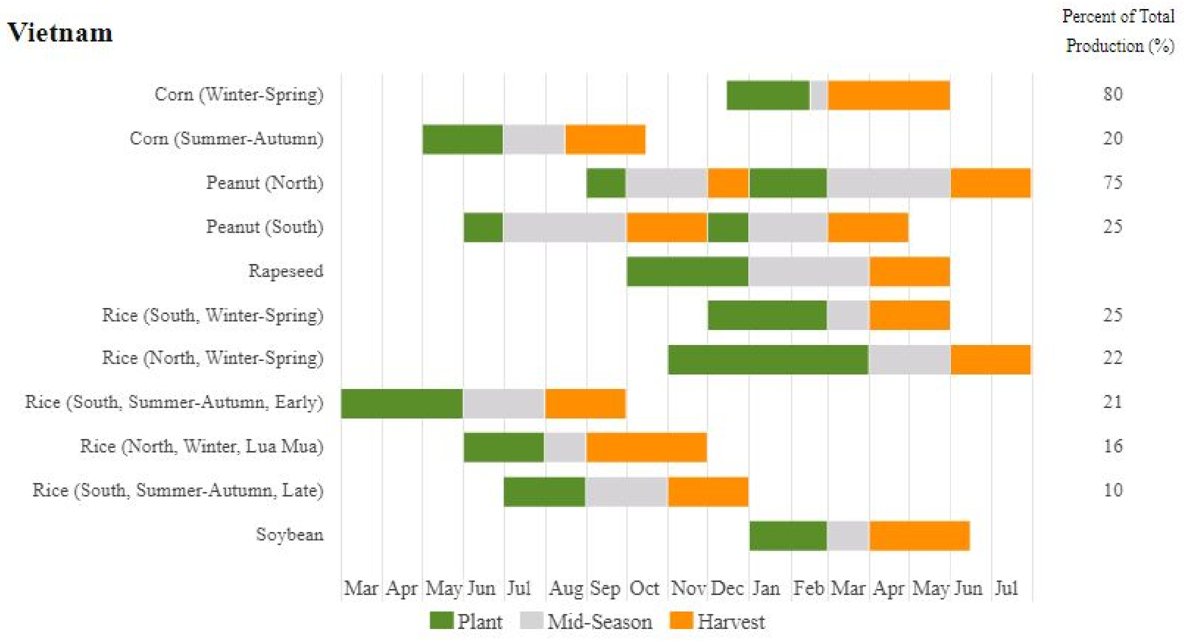

Figure S1. Crop calendars in Vietnam provided by USDA.

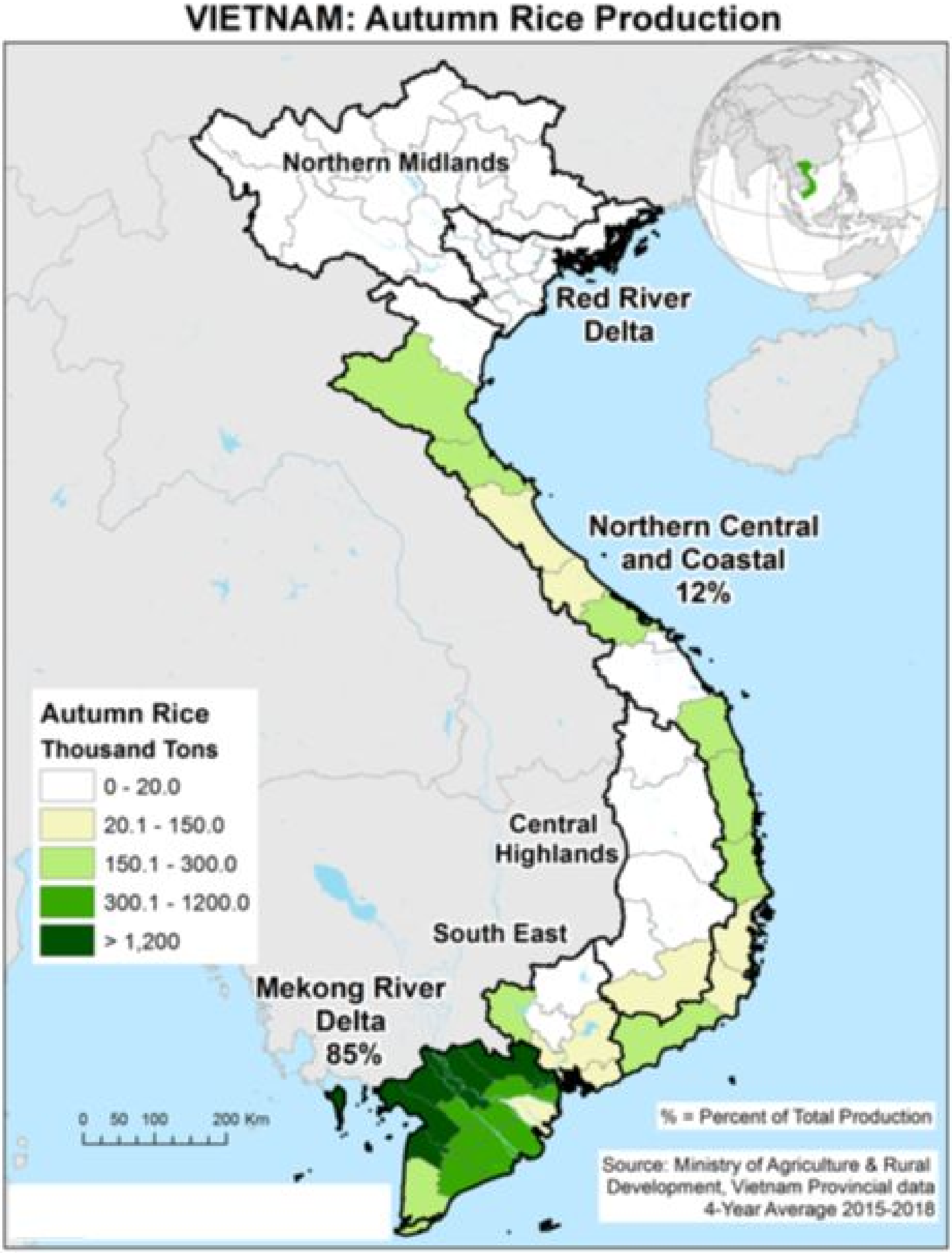

Figure S2. Distribution of autumn rice production provided by USDA. Note: This map is an image of country, not the actual border of land.

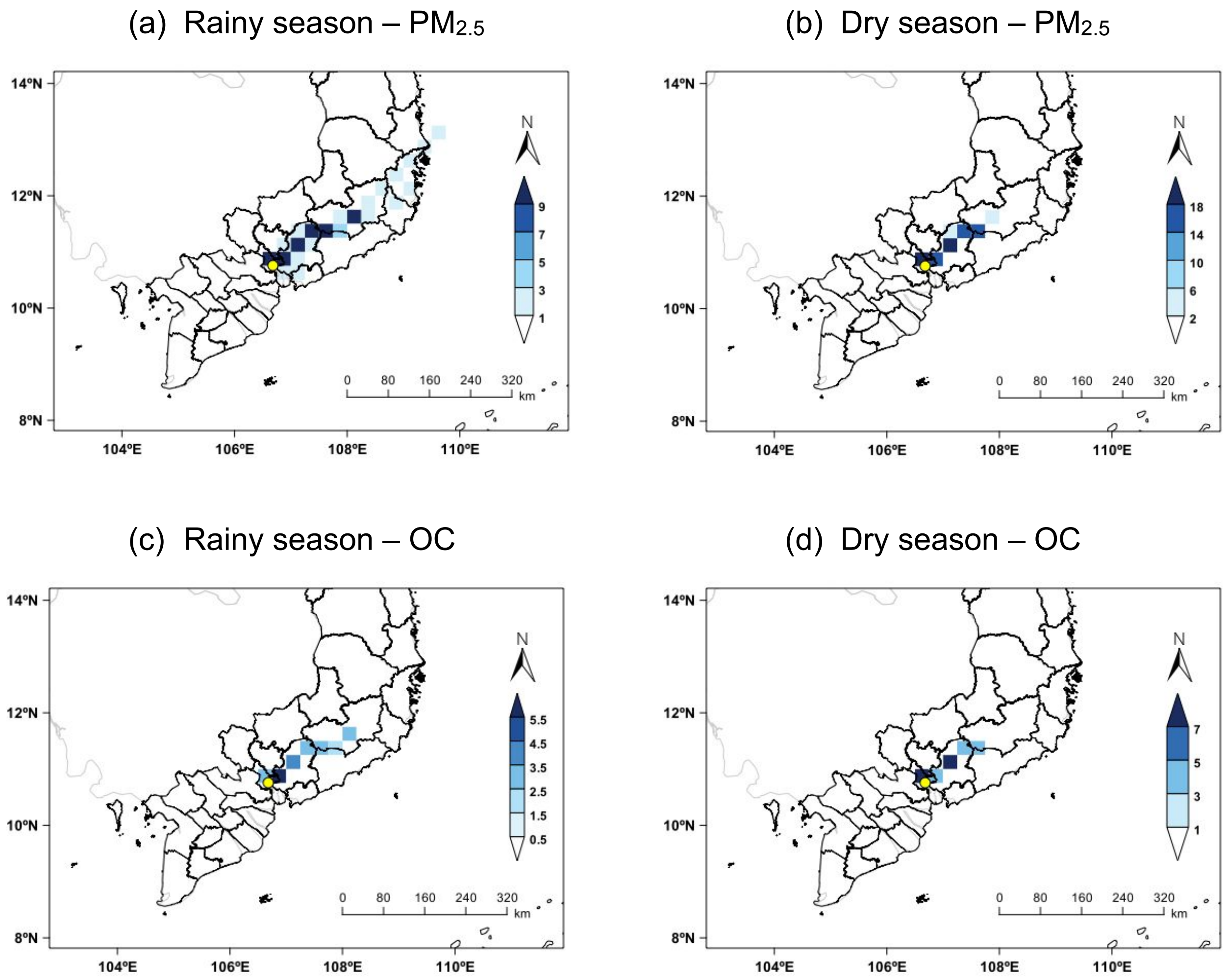

Figure S3. WCWT analyses of $PM_{2.5}$ (a–b), OC (c–d), K^{+}_{BB} (e–f), and Lev (g–h) in the rainy season (left) and dry season (right). Blue bars indicate the unit of $PM_{2.5}$, OC, and K^{+}_{BB} in μg m^{-3} and Lev in ng m^{-3}. Yellow dot represents the sampling site. Note: This map is an image of country, not the actual border of land.

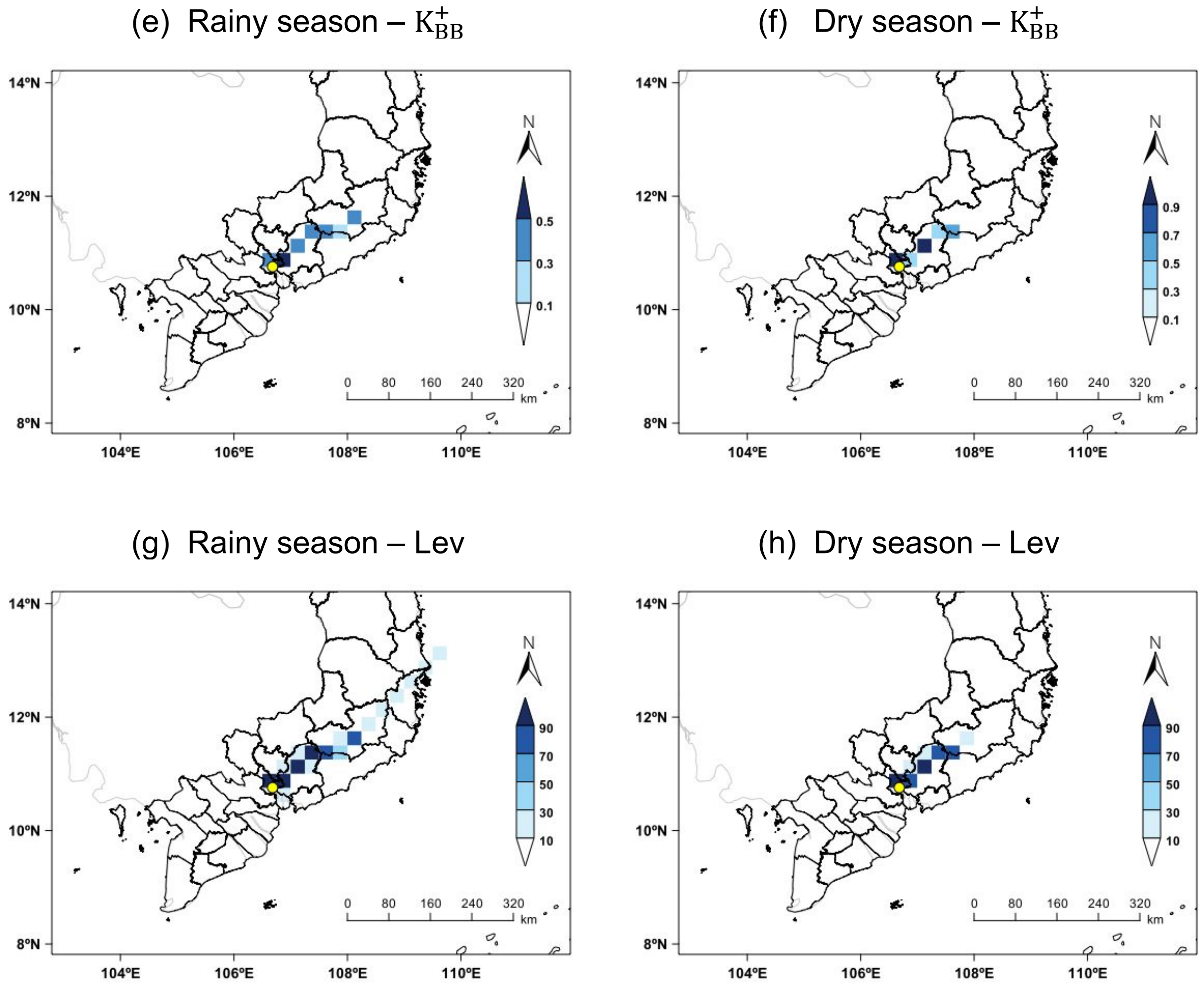

Figure S3. Continued
